# A Pilot Project to Introduce the Compassionate Approach to Living Mindfully for Prevention of Disease (Calmpod) in Weight Management in a Forensic Intellectual Disability Unit

**DOI:** 10.1192/bjo.2022.268

**Published:** 2022-06-20

**Authors:** Ayomipo Amiola, Phil Temple, Helen Dickerson, Peter Langdon, Petra Hanson, Thoral Thomas, Reena Tharian, Thomas Barber, Vinod Menon, Regi Alexander

**Affiliations:** 1 Hertfordshire Partnership University NHS Foundation Trust, Norwich, United Kingdom; 2 University of Warwick, Warwick, United Kingdom; 3 Norfolk and Suffolk Foundation Trust, Norwich, United Kingdom; 4 University Hospitals of Coventry NHS Trust, Warwick, United Kingdom; 5 University of Hertfordshire, Hatfield, United Kingdom

## Abstract

**Aims:**

About 28% of the UK population are obese and a further 36.2% are overweight. The prevalence of both in those with mental illness and/or intellectual disability (ID) is much higher. Several therapeutic approaches have been tried, with varying efficacy. Recently a three-session intervention which uses mindfulness techniques (The compassionate approach to living mindfully for prevention of disease- CALMPOD) was used in a tertiary obesity service in the West Midlands and shown significant benefits. Our aim was to assess the suitability of this intervention in mental illnesses and/or intellectual disability services.

**Methods:**

Three pre-pilot focus group discussions involving multispecialty professionals and service users were held involving four psychiatrists, three service users, two psychologists, one physician, one endocrinologist, one bariatric surgeon and one pharmacist to identify key aspects of the CALMPOD programme for adaption to psychiatry and/or psychiatry of ID wards. Based on this, CALMPOD was modified by two psychologists with relevant experience. The modified CALMPOD was piloted in a medium secure forensic in-patient unit for people with ID. A post-pilot focus group discussion involving two psychiatrists, one occupational therapist and three service users was held after completion of the pilot to discuss lessons learned.

**Results:**

Invitations sent to 17 in-patients. The mean BMI was 34.76%, 76% were obese, 6% were over-weight and 18% in the normal range of weight. 3 patients attended the three-session programme (17%). All 3 were in the obese category, all had had individual weight management input – i.e. seen by dietician, weight management included in care plans. The post-pilot focus group discussions identified 6 key themes.

**Conclusion:**

Emerging themes from the pilot were (a) Patients and staff recognise that the programme was ‘necessary’ and ‘useful’, but the challenge is how to ‘start attending regularly’. Once in, participants ‘tended to stay on’. (b) A visible publicity campaign is needed to spread awareness of the programme and its ‘newness’. This would help with staff ‘buy in’ from all wards and departments. (c) The key message should be ‘living healthily’ and ‘feeling better’, not just weight loss. (d) Staff and/or patients’ family members participating in the programme would be more motivating. (e) The content of the programme needs further modifying with an emphasis on shared activities, calories counting and less emphasis on definitions. (f)Calorie counts and exercise trackers need ‘more fun and interactive elements.

Based on these recommendations a revised CALMPOD- ID programme, co-produced with service users, is now being introduced in the service.

